# Economic evaluations of lymphatic filariasis interventions: a systematic review and research needs

**DOI:** 10.1186/s13071-018-2616-z

**Published:** 2018-02-01

**Authors:** Lukyn M. Gedge, Alison A. Bettis, Mark H. Bradley, T. Déirdre Hollingsworth, Hugo C. Turner

**Affiliations:** 1 School of Public Health, Faculty of Medicine, St Marys Campus, Imperial College London, Norfolk Place, London, W2 1PG UK; 2 London Centre for Neglected Tropical Disease Research, London, UK; 3 Department of Infectious Disease Epidemiology, School of Public Health, Faculty of Medicine, St Marys Campus, Imperial College London, Norfolk Place, London, W2 1PG UK; 4 Global Health Programs, GlaxoSmithKline, Brentford, UK; 5 Mathematics Institute, University of Warwick, Coventry, CV4 7AL UK; 6 School of Life Sciences, University of Warwick, Coventry, CV4 7AL UK; 7 Big Data Institute, University of Oxford, Oxford, OX3 7LF UK; 8 Oxford University Clinical Research Unit, Wellcome Trust Major Overseas Programme, Ho Chi Minh City, Vietnam; 9 Centre for Tropical Medicine and Global Health, Nuffield Department of Medicine, University of Oxford, Oxford, UK

**Keywords:** Lymphatic filariasis, Cost-benefit, Cost-effectiveness, Economic evaluations, Economic impact, GPELF, Programme evaluation

## Abstract

In 2000, the World Health Organization established the Global Programme to Eliminate Lymphatic Filariasis (GPELF), with the goal of eliminating the disease as a public health problem by 2020. Since the start of the programme, a cumulative total of 6.2 billion treatments have been delivered to affected populations - with more than 556 million people treated in 2015 alone. In this paper, we perform a rigorous systematic review of the economic evaluations of lymphatic filariasis interventions have been conducted. We demonstrate that the standard interventions to control lymphatic filariasis are consistently found to be highly cost-effective. This finding has important implications for advocacy groups and potential funders. However, there are several important inconsistencies and research gaps that need to be addressed as we move forward towards the 2020 elimination goals. One of the most important identified research gaps was a lack of evaluation of new interventions specifically targeting areas co-endemic with onchocerciasis and *Loa loa* - which could become a major barrier to achieving elimination.

**Electronic supplementary material:**

The online version of this article (10.1186/s13071-018-2616-z) contains supplementary material, which is available to authorized users.

## Background

Lymphatic filariasis (LF), is a human disease caused by parasitic helminths (*Wuchereria bancrofti*, *Brugia malayi* and *Brugia timori*). These filarial worms are transmitted via infected mosquitoes.

There are 73 endemic countries at-risk of LF, and before widespread control approximately 120 million people worldwide were infected - of whom 40 million were suffering from overt clinical disease [1, 2]. Clinical disease can manifest as painful severe swelling due to lymphedema (an accumulation of lymphatic fluid generally in the limbs), hydrocele (fluid accumulation in the scrotal sac) and episodes of acute adenolymphangitis [1, 2].

In 1997, the World Health Assembly passed Resolution 50.29, calling for the elimination of LF as a public health problem [3]. Following on from this, in 2000 the World Health Organization (WHO) established the Global Programme to Eliminate Lymphatic Filariasis (GPELF) with the goal of eliminating the disease as a public health problem by 2020 [4, 5]. The programme has two parallel goals [4, 5]:

(i) To use community-wide annual mass drug administration (MDA) to interrupt transmission, using a combination of albendazole and ivermectin in areas co-endemic with onchocerciasis, and albendazole and diethylcarbamazine (DEC) elsewhere.
(ii) To alleviate suffering by managing morbidity and preventing disability in clinical LF patients.

These goals are supported by the WHO’s 2020 Neglected Tropical Disease (NTD) Road Map [6] and the London Declaration on NTDs [7].

Some countries are acknowledged as having eliminated LF as a public health problem [8]*.* However, it is recognised that we are not currently on track to meet these goals in many settings, and achieving elimination may require alternative approaches [9–11].

One particular challenge facing LF elimination efforts in Africa is areas co-endemic with onchocerciasis and the tropical eye worm *Loa loa* (which causes loiasis). Traditionally, onchocerciasis is managed with annual or biannual (twice yearly) ivermectin treatment. However, due to the potential for severe and often fatal encephalopathic reactions to ivermectin in patients with high *L. loa* microfilaria loads, this therapeutic approach is not permissible in many loiasis co-endemic areas [12]. To facilitate LF elimination in these problematic co-endemic zones of central Africa, the WHO has proposed an alternative strategy that involves biannual albendazole monotherapy together with the expanded use of bed nets [13]. It is also important to restate that DEC can cause severe adverse reactions in individuals with heavy *Onchocerca volvulus* infections and that it is not used in onchocerciasis-endemic areas [14, 15].

As we move forward towards elimination, we need to better understand the cost-effectiveness of both the current and the potential alternative control strategies. The aim of this paper is to provide a systematic review of economic evaluations which have already been conducted for LF interventions and to summarise the key knowledge and research gaps in this area.

## Systematic review

### Search strategy and methodology

A systematic review of the literature was conducted in December 2016 using the PubMed (MEDLINE) and ISI Web of Science electronic databases. Variants of the following search terms were used to find relevant papers: lymphatic filariasis, cost(s), cost-benefit, cost-effectiveness, economic(s), economic evaluation. No date or language stipulations were applied to the searches. A more detailed summary of the search terms and the PRISMA checklist are supplied in Additional file 1.

**Fig. 1:**
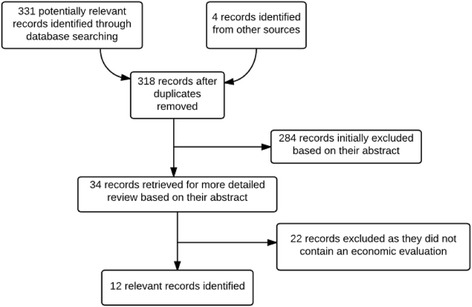
Decision tree outlining the inclusion and exclusion of the identified studies. Some studies reported both cost-benefit and cost-effectiveness estimates*.* Several ‘grey literature’ texts (including policy reports) which were not found within the databases, were also identified (using Google Scholar and the bibliographies of other papers). A PRISMA checklist is provided in Additional file 1

The titles and abstracts of all the identified papers were examined initially for relevance and then the bibliographies of papers suitable for inclusion were scanned for studies not originally retrieved from the databases. The full selection process is outlined in Fig. 1. This process was performed in duplicate.

### Summary of the identified studies

We identified 12 different primary sources reporting the results of economic evaluations of LF interventions. A summary of the studies is presented in Tables 1, 2. The majority of the estimates were evaluating MDA, though it was not always clear which drug combination was being investigated. Only two studies were identified that investigated the cost-effectiveness or cost-benefit of morbidity management strategies (Tables 1, 2).

**Table 1:** Summary of the identified cost-effectiveness analyses

Study	Research question	Study region	Time horizon	Intervention	Effectiveness metrics	Primary conclusions	Cost sources
Standard interventions							
[20]	The incremental cost-effectiveness associated with different intensities of scaling-up annual MDA coverage within the GPELF	Global	50 years	Three different rates of scaling-up the MDA coverage of the GPELF (Erad1, Erad2, Erad3-see legend)	DALYs averted	• The faster the coverage of the MDA programmes is scaled up, the greater the health gains and cost-effectiveness of the GPELF	^b^
						• This analysis suggests that more intense forms of scale-up are most likely to be cost-effective, lending further support to intensifying LF elimination efforts:	
						• Erad1 scenario^a^: US$ 219 (95% CrI: 142.65–322.72) per incremental DALY averted	
						• Erad2 scenario: US$ 120.7 (95% CrI: 79.47–177.70) per incremental DALY averted	
						• Erad3 scenario: US$ 72.94 (95% CrI: 47.74–109.80) per incremental DALY averted	
						*•* Costs are in 2012 US$	
[36]	Estimating an infection threshold that achieves control of LF-related disease	Tanzania	Not explicitly stated	Annual MDA for 5 (control) *vs* 10 years (elimination)	Prevalent cases cured	• A prevalence of microfilarial infection below a threshold of approximately 3.55%^c^ could constitute an achievable and sustainable target to control LF related disease	[94, 95]
						• Due to the high marginal cost of curing the last few individuals for elimination, the maximal benefits of LF control can occur at this threshold	
						*•* Cost year not clearly stated	
[2]	A preliminary cost-effectiveness estimate of the MDA provided by the GPELF (2000–2007)	Global	Lifetime of the benefit cohort resulting from the MDA provided between 2000 and 2007	Annual MDA	DALYs averted	• Assuming a treatment cost of US$ 0.10 per person would result in a cost per DALY averted of US$ 5.90	na
[16]	Cost-effectiveness of annual MDA	Based on data from India	30 years	Annual MDA (Control, Elim1, Elim2 - see legend)	DALYs averted	• It was estimated that in high prevalence areas, achieving elimination with MDA is highly cost-effective	Not explicitly stated
						• Even if elimination is not achieved and the treatment programme is continued for 30 years, MDA would still be considered highly cost-effective:	
						• Control scenario: US$ 29 per DALY averted	
						• Elim1 scenario: US$ 4.40 per DALY averted	
						• Elim2 scenario: US$ 8.10 per DALY averted	
						*•* Cost year not clearly stated	
[17]	Cost-effectiveness of the MDA provided by the GPELF (2000–2014)	Global	Lifetime of the benefit cohort resulting from the MDA provided between 2000 and 2014	Annual MDA	DALYs averted	• The projected cost-effectiveness of MDA was high and robust over a wide range of costs and assumptions:	[53]
						• Using financial costs: US$ 24 (12–39) per DALY averted	
						• Using economic costs excluding the donated drugs value: US$ 29 (14–48) per DALY averted	
						• Using economic costs including the donated drugs value: US$ 64 (49–83) per DALY averted	
						• The range is based on the predicted 95% confidence intervals for the treatment delivery costs	
						*•* Costs are in 2014 US$	
[17]	A preliminary cost-effectiveness analysis of a hydrocelectomy	Global	Lifetime of an average hydrocele patient	Hydrocele surgery	DALYs averted	• Under the health care provider’s perspective, it was projected that hydrocelectomy would be classed as highly cost-effective if the surgery cost < US$ 66, and cost-effective if < US$ 398 (based on the World Bank’s cost-effectiveness thresholds for low-income countries [18])	[96]
						• When using the societal perspective (which also includes the patients’ costs-such as for transportation and from lost wages) these results changed to US$ 29 and US$ 361, respectively	
						*•* Costs are in 2014 US$	
Alternative interventions							
[35]	How increasing MDA frequency to twice per year could affect the treatment programmes duration and total cost	India & West Africa	Up to 20 treatment rounds	Biannual (twice a year) *vs* annual MDA	Programme duration and total cost	• Model predictions suggested in most scenarios a biannual MDA strategy would require the same number of treatment rounds to achieve LF elimination as an annual MDA strategy	India: [97], West, Africa: [47]
						• Thus, biannual MDA programmes should achieve elimination in half of the time	
						• When excluding the economic value of the donated drugs the total programme costs for biannual MDA were projected to be lower in most scenarios	
						• When including the value of the donated drugs, biannual MDA remained the cheaper strategy in most of the Indian scenarios, but became slightly more expensive in the West African scenarios	
						• Costs are in 2009 US$	
[16]	Cost-effectiveness of vector control	Based on data from India	30 years	Vector control (Control, Elim1, Elim2 - see legend)	DALYs averted	• Control scenario: US$ 302.50 per DALY averted	Not explicitly stated
						• Elim1 scenario: US$ 47.50 per DALY averted	
						• Elim2 scenario: US$ 84.30 per DALY averted	
						• Cost year not clearly stated	
[16]	Cost-effectiveness of DEC-fortified salt	Based on data from India	30 years	DEC-fortified salt (Control, Elim1, Elim2 - see legend)	DALYs averted	• Control scenario: US$ 46.48 per DALY averted	Not explicitly stated
						• Elim1 scenario: US$ 1.10 per DALY averted	
						• Elim2 scenario: US$ 3.62 per DALY averted	
						• Cost year not clearly stated	
[94]	The cost-effectiveness of four different mass DEC chemotherapy regimens	Tanzania	2 years	(i) Standard dose daily for 12 days	Prevalent cases cured	• The most cost-effective strategy was found to be the low monthly dose of DEC treatment	Presented in the same paper
				(ii) Biannual standard doses for a year		• However, the sensitivity analyses indicated that the optimal choice of DEC strategy was sensitive to the assumed programme design	
				(iii) Low dose given monthly for a year		• The results suggested that if the delivery structure was simplified, DEC-medicated cooking salt had the potential to be the dominant intervention	
				(iv) Distributing DEC-fortified salt for a year		• Costs are in 1995 US$	
[37]	Cost-effectiveness analysis of using a combination of both vector control and MDA	India	5 years	Combination of 2 annual rounds of MDA and vector control activities (lasting 3 years) *vs* 2 annual rounds of MDA alone	(i) Infective bites prevented	• Integration of vector control with MDA did not appear to be cost-effective in this setting	Presented in the same paper
						• MDA alone:	
						• Cost per infective larva prevented: US $3.14	
						• Cost to reduce microfilarial prevalence by 1%: US$ 96.62	
						• Combination of vector control and MDA:	
						• Incremental cost per additional infective larva prevented: US$ 16.32	
						• Incremental cost per additional 1% reduction in microfilarial prevalence: US$ 1451.97	
					(ii) Infective larvae prevented	• Incremental cost of stopping each additional infective bite/villager: US$ 46.92	
					(iii) Prevalence averted	*•* Costs are in 1997 US$	

*Abbreviations*: *CrI* credible interval, *DALYs* disability-adjusted life years, *DEC* diethylcarbamazine, *GPELF* Global Programme to Eliminate Lymphatic Filariasis, *LF* lymphatic filariasis, *MDA* mass drug administration, *na* not applicable

^a^Measured against the elimination scenario as the comparator (mirroring the current rate of MDA scale-up, but assuming that the countries that have not yet begun MDA programmes will not do so)

^b^Manuscript in preparation at the time of that publication

^c^Blood sampling volume of 1 ml

Erad1; expanding annual MDA to all endemic areas at the historical average rate of scale-up, Erad2; countries scale-up geographic coverage of annual MDA by 20% increments each year, Erad3; All countries expand coverage of annual MDA to their entire at-risk population immediately. Control; transmission is brought to low levels but not interrupted and where control efforts will have to continue (for the full-time horizon). Elim1; sustained interruption of transmission is achieved after a short period of intervention (6 years of annual MDA or 10 years of vector control or 2 years of DEC-fortified salt). Elim2; sustained interruption of transmission is achieved after a longer period of intervention (10 years of annual MDA or 15 years of vector control or 4 years of DEC-fortified salt)

**Table 2:** Summary of the identified cost-benefit analyses and estimates of the economic benefits of interventions

Study	Research question	Study region	Time horizon	Intervention	Outcomes	Primary conclusions	Cost sources
Economic benefits of interventions							
[20]	The economic benefits associated with different rates of scaling-up MDA within the GPELF	Global	50 years	Three different rates of scaling-up the MDA coverage of the GPELF	(i) Prevented potential productivity/income losses	• Extending coverage to all LF endemic areas could generate additional economic benefits through potential gains in worker productivity between US$ 3.4 billion and US$ 14.4 billion and could result in health systems savings of up to US$ 483 million due to averted morbidity management costs.	(i) [98]; (ii) [21, 99]
					(ii) Prevented costs to the health system for caring for clinical patients		
						• Costs are in 2012 US$	
[21]	The economic benefit resulting from the MDA provided by the GPELF (2000–2007)	Global	Lifetime of the benefit cohort resulting from the MDA provided between 2000 and 2007	Annual MDA	(i) Prevented medical expenses incurred by patients	• An estimated US$ 24 billion in potential economic benefits will be gained over the lifetime of those treated by the GPELF between 2000 and 2007	(i)^a^; (ii)^b^; (iii) [99]
					(ii) Prevented potential productivity/income losses	• This total amount results from summing the estimated prevented medical expenses incurred by LF patients (US$ 1.4 billion), prevented potential productivity/income losses (US$ 20.4 billion), and prevented costs to the health system (US$ 2.2 billion)	
					(iii) Prevented costs to the health system resulting for clinical patients		
						*•* Costs are in 2005 US$	
[22]	The economic benefit resulting from the MDA provided by the GPELF (2000–2014)	Global	Lifetime of the benefit cohort resulting from the MDA provided between 2000 and 2014	Annual MDA	(i) Prevented medical expenses incurred by patients	• An estimated US$ 100.5 billion in potential economic benefits will be gained over the lifetime of those treated by the GPELF between 2000 and 2014 and 36 million clinical LF cases will be averted	(i)^a^; (ii)^b^; (iii) [21, 99]
					(ii) Prevented potential productivity/income losses	• This total amount results from summing the estimated prevented medical expenses incurred by LF patients (US$ 3 billion), prevented potential productivity/income losses (US$ 94 billion), and prevented costs to the health system (US$ 3.5 billion)	
					(iii) Prevented costs to the health system resulting for clinical patients		
						• The average lifetime economic benefit to an individual with averted clinical disease was estimated to be US$ 2095	
						*•* Costs are in 2014 US$	
[100]	The economic benefit of MDA in India	India	11 years (based on the average number of years of productive life lost)	Annual MDA	(i) Prevented medical expenses incurred by patients	• The economic benefit accrued by averting a chronic case was projected to be US$ 40.83 per year	(i) [28]; (ii) [28]
					(ii) Prevented potential productivity/income losses	• This included preventing US$39.39 in potential productivity/income losses each year (58.24 working days) and US$ 1.44 in prevented medical expenses	
						• It was estimated that chronic disease afflicts patients for an average of 11 years of productive life and the total lifetime economic benefit was estimated to be US$ 449.13 per chronic case averted	
						*•* Cost year not clearly stated	
[23]	Economic benefits of community-based lymphedema management	India	Productive working lifetime of lymphedema patients projected over a 60-year period	Lymphedema Management	(i) Prevented medical expenses incurred by patients	• The estimated long-term economic benefit of the investigated lymphedema management programme was US$ 26.1 million	(i) [101, 102]; (ii) [103]
					(ii) Prevented potential productivity/income losses	• This corresponds to an average benefit of US$ 1648 per participant of working age (equivalent to 1258 days of earnings over their lifetime)	
						• Real wages and real expenditure on medical care were assumed to rise 4% per year	
						*•* Costs are in 2008 US$	
Cost-benefit analysis of interventions							
[21]	The cost-benefit of the MDA provided by the GPELF (2000–2007)	Global	Lifetime of the benefit cohort resulting from the MDA provided between 2000 and 2007	Annual MDA	Benefit-cost ratio	• The study estimated country-specific benefit-cost ratios for years of the GPELF with corresponding treatment cost data [47]	[47]
						• Results ranged between 1.64–18.07 when using financial costs, and 0.21–8.59 when using the economic costs (including the donated drugs value)	
						• The ratios were lower in settings where ivermectin was used (due to its higher economic value)	
						*•* Costs are in 2005 US$	
[17]	The cost-benefit of the MDA provided by the GPELF (2000–2014)	Global	Lifetime of the benefit cohort resulting from the MDA provided between 2000 and 2014	Annual MDA	Benefit-cost ratio	• The benefit-cost ratios varied depending on what costs were included in the analysis:	[53]
						• Using financial costs: 36 (23–74)	
						• Using economic costs- excluding the donated drugs value: 30 (18–63)	
						• Using economic costs- including the donated drugs value: 14 (11–18)	
						• The range is based on the predicted 95% confidence intervals for the treatment delivery costs	
						• Costs are in 2014 US$	
[100]	The cost-benefit of MDA in India	India	11 years for the economic benefits and 6 years for the intervention costs	Annual MDA	Benefit-cost ratio	• Estimated that preventing a chronic LF case has a benefit-cost ratio of 53.4 (not discounted)	[97]
						• This is based on an estimated economic benefit of US$ 449.13 per chronic case averted and assumes that the prevention of 1 chronic case (through 6 MDA rounds) costs US$ 8.41	
						• Cost year not clearly stated	
[23]	The cost-benefit of community-based lymphedema management	India	Productive working lifetime of lymphedema patients projected over a 60-year period	Lymphedema management	Benefit-cost ratio	• To implement/operate the community-based lymphedema management programme for 2 years cost between US$ 10.00–12.50 per person [104]	[104]
						• An average participant can expect lifetime economic benefits 132–165 times greater than the per-person cost of the programme	
						*•* Costs are in 2008 US$	

*Abbreviations*: *Cr* credible interval, *GPELF* Global Programme to Eliminate Lymphatic Filariasis, *LF* lymphatic filariasis, *MDA* mass drug administration

^a^Estimated within the paper (based on the approach taken in [21])

^b^The lowest of the four different wage sources (based on the approach taken in [21])

Due to the different aims of the identified studies, a variety of different effectiveness measures were used by the different analyses - including the cost to elimination, cost per disability-adjusted life year (DALY) averted, the benefit-cost ratio, the cost per case cured. Several studies [2, 16, 17] used DALYs averted as the effectiveness measure to quantify the health impact of MDA - therefore their outcomes are directly comparable to each other. The cost-effectiveness ratios varied depending on which costs were included and the time horizon of the analysis (Table 1). However, they all would class MDA for LF as either cost-effective or highly cost-effective based on the thresholds for low-income countries established by the World Bank (≤ US$ 251 per DALY averted = cost-effective [18], and ≤ US$ 42 per DALY = highly cost-effective [18] (adjusting for inflation - 2016 prices) [19]). Stone et al. [20] also used DALYs averted as an effectiveness metric, and estimated the incremental cost-effectiveness of three different scenarios for accelerating the rate of MDA coverage scale-up (Table 1). Within this study, they also estimated the savings to the health system and the gains in worker productivity (Table 2).

Chu et al. [21] and Turner et al. [22] projected that the MDA provided under the GPELF would result in substantial economic benefits. The clear majority (> 80%) of this estimated economic benefit resulted from the prevention of the potential productivity/income losses associated with LF morbidity (indirect costs, Table 3). These studies were based on the same framework, and an explanation for the differences in the results is outlined in Turner et al. [22]. Stillwaggon et al. [23] also found notable economic benefits and productivity gains resulting from a community-based lymphedema management programme in India (Table 2).

**Table 3:** Glossary

Term	Definition
Benefit-cost ratio (BCR)	The ratio of the monetary benefits of an intervention relative to its costs.
Cost-effectiveness ratio	A statistic used to summarise the cost-effectiveness of a health care intervention. It is defined as the cost of an intervention, divided by its effectiveness.
Direct costs	Direct costs represent the value of the goods, services, and resources consumed in providing and accessing health care. These can be split into two types: the costs borne by the health system (such as for personnel and hospital services), and the costs borne by the patients/the community (such as for transportation to the health facility).
Disability-adjusted life years (DALYs)	DALYs are a measure of disease burden and are calculated as the sum of the years of life lost due to premature mortality and the years of healthy life lost due to disability. The number of years of healthy life lost due to disability are calculated using a disability weight factor (which is between 0 and 1) that reflects the severity of the disease. One DALY can be thought of as one year of “healthy” life lost.
Discounting/discount rate	Discounting is the process of adjusting future costs and outcomes to a “present value”. The discount rate determines the strength of the time preference.
Economic costs	Economic costs represent the full value of all the resources used for an intervention – including the value of donated resources. These are important when considering issues related to the sustainability and replicability of interventions. Examples of resources, which often have no financial costs but can have important economic costs are the ‘free’ use of building space provided by Ministries of Health, and the time devoted to mass drug administration by volunteer community drug distributors.
Economies of scale	The reduction in the average cost per unit resulting from increased production/output: in this case, the reduction in the cost per treatment as a result of increasing the number treated.
Economies of scope	The reduction in the average cost per unit resulting from producing two or more products at once: in this case, the reduction in the cost per treatment, when delivering more than one intervention at once (i.e. integrated control programmes)
Financial costs	The actual expenditure (i.e. the amount paid) for the goods and services that are purchased.
Fixed costs	Costs that are not dependent on the amount of output: in this case costs that do not change regardless of the total number of people treated.
Friction cost approach	The friction cost approach takes the employer’s perspective for valuing lost productivity, and therefore only counts as lost, the hours not worked before another employee takes over the patient’s work [32, 33]. It is based on the assumption that an ill individual can eventually be replaced by a healthy worker - therefore the initial productivity levels are restored after this ‘friction period’.
Human capital approach	The human capital approach takes the patient’s perspective for valuing lost productivity and therefore counts any hour not worked by the patient as an hour lost. With this approach, all potential production not performed by a patient because of morbidity or premature mortality is counted as a production loss [32].
Indirect costs (productivity costs):	Indirect costs represent the value of the productivity losses that result from illness, treatment, or premature death.
Perspective	The study perspective is the viewpoint from which the intervention’s costs and consequences are evaluated. When adopting the healthcare providers perspective, the costs falling outside the healthcare sector are ignored. In contrast, when adopting the societal perspective, all relevant cost categories should be included - including those incurred by the patients.
Time horizon	The time horizon for the analysis determines the duration over which the outcomes and costs are calculated.

Other studies have also highlighted the importance of the productivity losses associated with LF morbidity [24, 25]. For example, it has been estimated that in India, between 3.8–8.0% of the potential male labour input was being lost due to LF morbidity [26, 27] - subsequently valued at US$ 704 million per year (1995 prices) [28]. A similar value has been reported for Ghana, were over 7% of potential male labour was estimated to be lost due to chronic LF [29]. It is noteworthy that non-filarial elephantiasis (podoconiosis) has also been found to be associated with significant productivity losses [30].

It should be highlighted that these types of economic burden/benefit estimates are highly dependent on assumptions regarding the effect of clinical disease on productivity [21, 31], the number of years of productive life lived with clinical disease, and employment rates. In addition, when comparing these estimates, it is particularly important to consider which method and wage source has been used to value the productivity losses, as these can be highly variable even when referring to the same type of profession (highlighted in Additional file 1: Table S1). Furthermore, it is important to note whether lost wages were adjusted for future inflation or for future real wage growth (such as in [23]) as this could result in higher economic benefits/burden estimates. All of the studies that we found investigating the economic benefits resulting from LF interventions used the human capital approach to value the prevented productivity losses. This takes the patient’s perspective for valuing lost productivity and therefore counts any hour not worked by the patient as an hour lost - not accounting for the possibility that absent workers may be replaced (Table 3) [32]. It is worth noting that an alternative method known as the friction cost approach takes the employer’s perspective and therefore only counts as lost, the hours not worked before another employee takes over the patient’s work [32]. If this approach had been used, the estimated economic benefits could have been significantly lower [33]. There is continued debate regarding which approach is most appropriate [32]. Interestingly, the second US public health service panel on “cost-effectiveness in health and medicine” recently recommended using the human capital approach [34].

Only five cost-effectiveness estimates were identified which evaluated alternative interventions to the currently recommended strategies (outlined in Table 1). Furthermore, no studies were found that evaluated interventions specific for loiasis co-endemic areas.

The majority of the estimates had either no sensitivity analysis conducted or only univariate sensitivity analysis (where the impact of changing one parameter at a time is evaluated). The two main exceptions to this were Stone et al. [20] and Stolk et al. [35].

### The assumed costs of mass drug administration

#### Delivery costs

When comparing the different studies, it is important to consider that there is variation in the assumed delivery costs of MDA, even for estimates pertaining to the same country. The majority of the studies were based on the same relatively small number of costing studies (Tables 1, 2), and several of the cost-effectiveness/cost-benefit estimates were not based on published costing studies/data. This meant it was not always clear which costs were being included in the analyses, at times making it difficult to judge the generalizability of these studies.

It is also important to recognise whether or not the studies are using financial or economic cost data (Table 3). The following were the studies that clearly stated that they are using economic costs for the investigated intervention in at least a subset of the analysis [17, 20, 21, 35–37]. However, even in these cases it was not always clear which economic costs were being included. For example, the economic value of the volunteer community drug distributors’ time was not always included within the economic costs.

#### Drug costs

Depending on the perspective of the analysis, the value of the donated drugs may also be included as an economic cost. Several of the identified studies considered the economic value of the donated drugs within their economic evaluation - which increases the intervention’s cost (Table 4) and therefore decreases the estimated cost-effectiveness/cost-benefit (Table 1). However, it is important to note that there was variation in the assumed economic value of the drugs, and in some cases the official figures have changed over time. For example, in 2009 GlaxoSmithKline changed their valuation of donated albendazole to US$ 0.045 per tablet from $0.19 per tablet (GSK, unpublished) [38]. A summary of the economic value of the drugs assumed by Turner et al. [17] is outlined in Table 5.

**Table 4:** Summary of the average treatment costs of the GPELF (2000–2014)

Cost type	Average cost per treatment (95% CI)
Financial costs	US$ 0.46 (0.21–0.76)
Economic costs - excluding the donated drugs value	US$ 0.56 (0.25–0.94)
Economic costs - including the donated drugs value	US$ 1.32 (1.00–1.69)

*Notes*: The shown costs represent an overall average of the GPELF (2000–2014) adapted from Turner et al. [17]. The delivery costs were estimated using the web-based regression MDA costing model developed by the WHO [53]. It should be noted that model parametrisation relating to the use of paid health workers and not community volunteers for the drug distribution was used (resulting in a higher unit delivery cost). Further details are provided in Turner et al. [17]. Prices were adjusted to 2014 US$ [19]

**Table 5:** Drug costs and their economic value

Drug and dose	Average number of tablets needed per treatment^a^	Cost/value of each tablet (US$)	Shipping cost per tablet (US$)	Average cost/value per treatment (US$)^b^	Donation status
DEC (100 mg per tablet)	2.75 [35]	0.0144^c^	Included in the tablet cost estimate	0.044	Eisai: 2.2 billion DEC tablets to be donated by 2020 (achieved WHO pre-qualification in 2013).
Albendazole (400 mg per tablet)	1 [105]	0.045^d^ [38]	0.0019 [47]	0.052	GSK: 600 million albendazole tablets available for LF control annually until it is eliminated as a public health problem
Ivermectin (3 mg per tablet)	2.8 [106]	1.5^e^ [06]	0.005^e^ [106]	4.635	Merck & Co. Inc.: Unlimited supply for the treatment of onchocerciasis and LF for as long as needed

*Abbreviations*: *LF* lymphatic filariasis, *GSK* GlaxoSmithKline

^a^For DEC and ivermectin the number of required tablets per treatment is depended on the age or height of the recipient and therefore the overall average is not a whole number

^b^Includes a wastage factor of 10%

^c^Eisai, Unpublished

^d^GSK, Unpublished

^e^Mectizan Donation Program, Unpublished. It should be noted that these are the costs/values reported by the drugs companies that donate them. However, it is possible to procure the drugs at lower prices (see International Drug Price Indicator Guide (http://erc.msh.org/priceguide)). The table is adapted from Turner et al. [17]

Turner et al. [17] found that when only considering countries using the ivermectin and albendazole regimen, that the GPELF would no longer be classed as cost-effective when using the World Bank thresholds (although only marginally and it remained highly cost-effective based on the WHO-CHOICE thresholds [39]). This is due to the higher economic value of ivermectin (Table 5). Despite this result, the GPELF was found to be clearly cost-effective as a whole [17]. Stolk et al. [35] also found that including the value of the donated drugs, decreased the potential economic benefits of increasing the treatment frequency to twice a year. It should be noted that it is difficult to estimate the true economic value of these donated drugs [17]. Furthermore, it is important to consider that the foundation of the GPELF is based on the long-term and sustained commitment of drug donations of ivermectin and albendazole for as long as needed until the elimination of LF is achieved [40], and the majority of the required DEC is being donated up to 2020 (Table 5). It should also be noted that drug donations are the primary basis for many NTD MDA programmes.

### Limitations

A potential source of bias within this review is that the employed search strategy could not always retrieve economic evaluations outside of published papers (i.e. grey literature such as policy documents and reports). This bias was minimised by searching the bibliographies of selected studies and the use of Google Scholar. This resulted in four publications being added to the initial compilation.

It should be noted that there could be a degree of publication bias, with economic evaluations with negative or unfavorable results being less likely to be published.

## The cost-effectiveness of control *versus* elimination

**Fig. 2:**
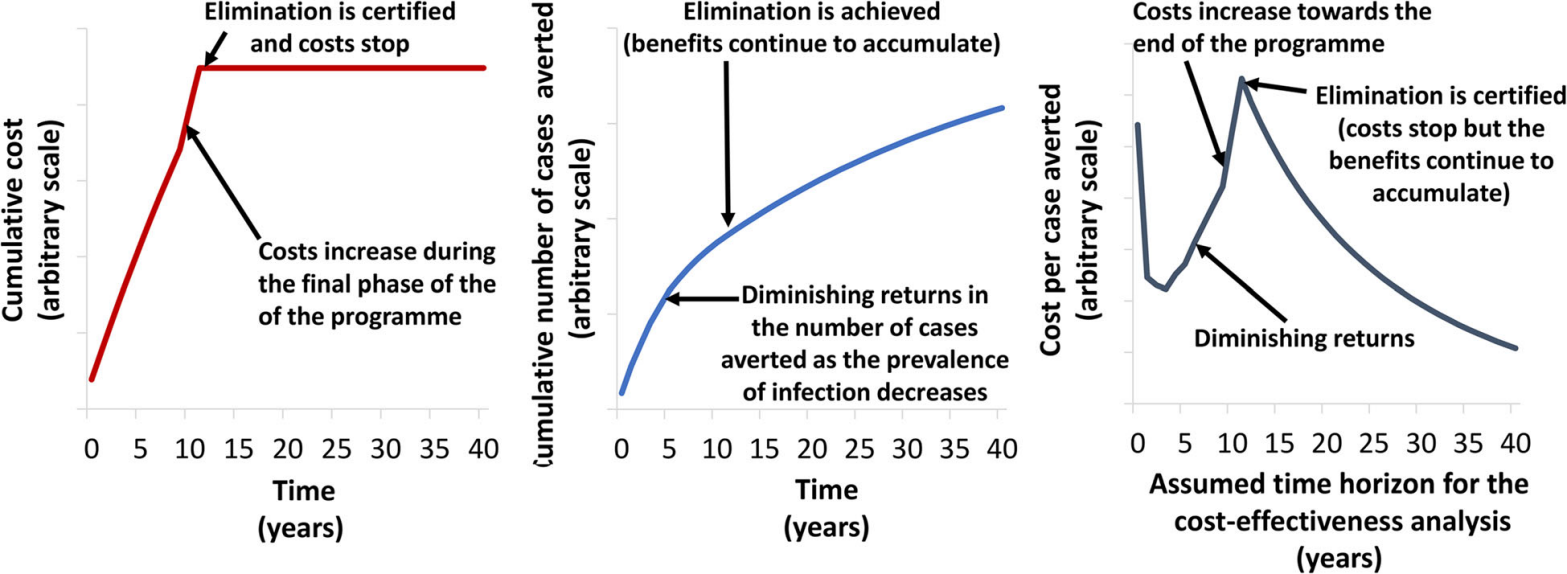
A theoretical diagram of the potential cost, effectiveness and cost-effectiveness of a mass drug administration programme before and after elimination*.* Note that this figure is illustrative and not based on primary data. The time horizon for the cost-effectiveness analysis is the duration over which the outcomes and costs are calculated. Both the cost and effects are being discounted into the future at a rate of 3%

When comparing the different studies, it is important to consider the time horizon used for the analysis and whether the study is evaluating morbidity control or the elimination of transmission. Michael et al. [36] found that a MDA programme’s cost per case cured can be higher when its aim is to eliminate transmission compared to when its aim is only morbidity control. The analysis highlighted that a MDA programme’s peak cost-effectiveness can occur at a point before full disease control is achieved. This is because, as the prevalence of infection decreases, the incremental cost per additional infection cured can increase steeply for each subsequent MDA round (illustrated in Fig. 2). However, depending on the time horizon and assumptions of the analysis, it is possible that an elimination campaign will become more cost-effective in the long-term and potentially even cost-saving (Fig. 2). For example, Remme et al. [16] found that with a 30-year time horizon, an elimination strategy would be more cost-effective than a morbidity control strategy (where transmission is brought to low levels but not interrupted). This was because, though an elimination strategy is more expensive to run, after elimination has been achieved, MDA and its associated costs stop. In contrast, for the control scenario, transmission is not broken so the costs associated with MDA are incurred for the full-time horizon (Table 1). Due to this, the control scenario ultimately has a higher total cost over the 30 year time horizon (even though it was initially cheaper). It is important to highlight that in these studies, the potential cost savings resulting from achieving elimination/eradication are not infinite [20, 41], as the costs being considered are restricted within the study’s time horizon and are often discounted into the future.

These principles are highlighted in Fig. 2. In this hypothetical example, the cumulative cost of the programme steadily increases over time but then increases at a faster rate during the final phase of the programme - due to the costs associated with scaling-up into harder-to-reach areas, and the cost of the surveys needed to confirm the programme can be stopped, i.e. post-MDA surveillance. After elimination is certified, the cumulative costs stop increasing. In contrast, the cumulative effectiveness of the programme also increases over time, but shows a degree of diminishing returns (because as the intervention progresses fewer cases are prevented with each subsequent MDA round). As a result of these relationships, the cost-effectiveness of the programme is not constant and is highly dependent on the time horizon of the analysis. In this example, as the time horizon is increased, the cost-effectiveness will initially increase during the first phase of the programme but then start to decrease due to the diminishing returns in effectiveness (as the level of infection/transmission is reduced) and then decrease further when the costs rise during the final phase of the programme. After elimination is certified, the cost-effectiveness will steadily increase with the time horizon, as the costs have stopped but the benefits continue to accumulate (though they are discounted into the future). In this context, it is important to highlight that instantaneous cost-effectiveness ratios (i.e. comparing the costs and benefits at one selected time point) are not particularly informative, and it is the total cost and total effect for the assumed time horizon that should be evaluated.

It is noteworthy that alternative interventions aimed at accelerating and sustaining elimination may only have small “incremental health gains” but a large influence on the programme’s overall total cost (as seen for onchocerciasis [42]). In such cases, an incremental cost-effectiveness ratio in terms of the cost per additional DALY averted may not reflect the true value of these novel interventions. Kastner et al. [41] also highlighted that the number of DALYs averted may not be the best measure to assess the possible benefits of disease eradication - as the long-term consequences and broader benefits are not necessarily fully captured. A cost-benefit analysis may be more useful in capturing these benefits more fully.

## Areas that need further research

The results of the review indicate that the standard LF control strategies are consistently found to be cost-effective or cost-saving. However, there are some important inconsistencies and research gaps that need to be addressed as we move forward towards the 2020 goals, particularly regarding the evaluation of alternative elimination strategies.

In the following section we outline several key research needs.

### Settings co-endemic with loiasis

Due to the potential for life-threatening adverse events in intensely infected *L. loa* patients, alternative strategies to address the elimination of LF where loiasis is prevalent have been proposed [12]. In 2013, the Strategic and Technical Advisory Group for NTDs (STAG) recommended albendazole monotherapy combined with coordinated vector control in areas co-endemic with loiasis [13]. The impact of this albendazole monotherapy strategy is currently being evaluated in parts of central Africa [13, 43] as is a “Test-to-Exclude” from treatment approach [44]. However*,* none of the identified economic evaluations focused on strategies for these co-endemic areas, and policy for these settings is a notable research gap for LF elimination. This gap is not necessarily surprising, as currently the main objective and focus for these areas is still to find strategies that work and are safe.

It should be highlighted that the novel strategies (such as the “Test-to-Exclude” from treatment approach) in these settings could be more expensive than conventional MDA strategies. It will be important to consider the value of these interventions not only in reducing the burden in co-endemic areas, but also in their capacity to help enable the global elimination goals to be reached and the reduced risk that sustained transmission in these co-endemic settings results in the re-establishment of transmission in neighbouring areas.

It is important to consider that loiasis is a vector-borne disease (transmitted by *Chrysops* spp.) and another potential solution for these areas is to use vector control to reduce its transmission - reducing the overall burden of *L. loa* in these population and hence to risk of the severe adverse events associated with high microfilaria loads [45].

### Morbidity management strategies

A key element of the WHO’s strategy to combat LF involves increased morbidity management and disability prevention activities [4, 46]. However, we identified only two studies in this area - one on lymphedema management and one on hydrocele surgery (Tables 1, 2).

To allow for more economic evaluations of LF morbidity management strategies (across a range of settings), more data are urgently needed assessing their costs, resource requirements, clinical effectiveness, and the incidence of complications/relapse for the different potential techniques.

### Methodological issues and data needs

#### Treatment delivery costs

**Fig. 3:**
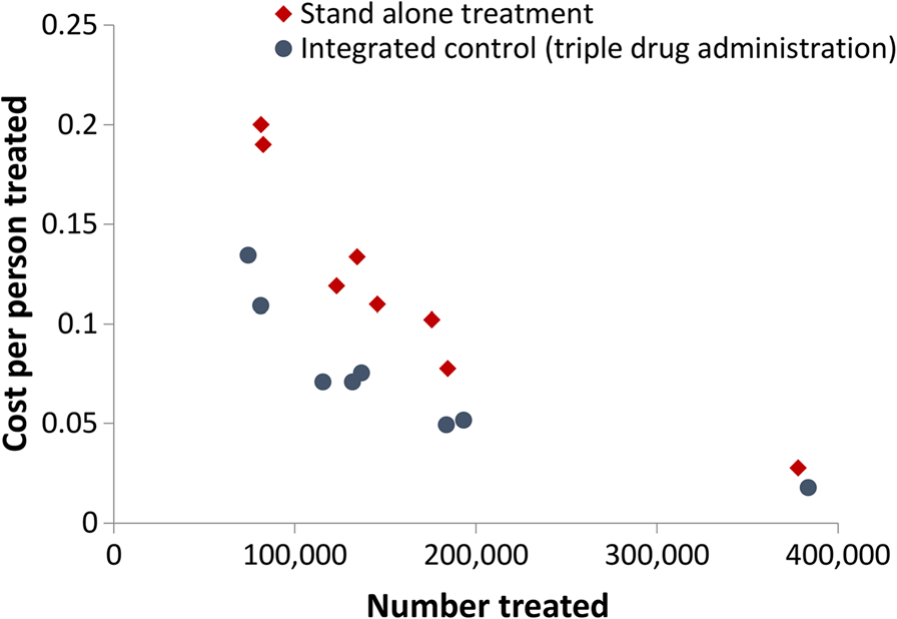
Observed economies of scale and scope associated with preventive chemotherapy. Data adapted from Evans et al. [51]. Costs are in 2008 and 2009 US$ prices

The costs of MDA delivery vary in different regions (highlighted by a multi-country costing study by Goldman et al. [47] and the systematic review by Keating et al. [24]). Understanding this variation and quantifying its impact is an important research gap for future studies - as it potentially affects the generalisability of cost-effectiveness/cost-benefit analysis [48]. In particular, one of the key drivers in the variation in delivery costs is the economies of scale associated with MDA [49–51] - the reduction in the cost per treatment as a result of increasing the scale of the programme (Fig. 3). However, the majority of studies identified in this systematic review assumed a constant cost per treatment and did not take into account the potential changes over time or scale (Tables 1, 2). The economies of scale associated with MDA are vital to consider when projecting the future costs of LF control, as well as when estimating the incremental costs of adopting alternative strategies. Furthermore, additional clarity regarding which costs are being included in the analysis will be important in future studies.

There are few costing studies investigating alternative strategies (such as increasing the treatment frequency [52]) [53]. In these cases, it is vital to consider the generalizability of the estimated difference in cost between the alternative and standard strategies across different programmatic settings. This is particularly significant if the costs of the alternative strategy have been estimated within a randomised control trial.

It should be noted that the unit delivery costs for the programmes will likely increase considerably as they approach the “last mile” towards elimination. This is because of the increase in the costs resulting from expanding the programmes to target harder-to-reach areas/groups (diseconomies of scale) and costs relating to conducting transmission assessment surveys (TAS). This has been seen in other interventions - particularly elimination campaigns [54–57]. Furthermore, it is important to note that as programmes start closing down implementation units, their costs will not decrease linearly (Fig. 3).

#### Programme integration

A notable research gap is the lack of understanding of the costs of integrated NTD control [24, 58] and how integration may influence the cost and cost-effectiveness of implementing different control strategies (economies of scope) (Fig. 3). Evans et al. [51] found that integrating MDA for LF with that for schistosomiasis, STH and onchocerciasis in Nigeria reduced the cost per treatment by 41% (not including the drug and overhead costs). The role and impact of this economies of scope should be considered further in future analyses.

#### Ancillary benefits of LF control programmes

The GPELF uses broad-spectrum antiparasitic drugs, and consequently, it has substantial auxiliary benefits on other parasitic diseases such as onchocerciasis, scabies, and the soil-transmitted helminths (STH) (described in more detail in [2, 22]). These auxiliary benefits are not typically included in economic evaluations of LF control programmes, which therefore underestimates their cost-effectiveness and cost-benefit. Furthermore, the end of LF-related MDA programmes is likely to have a considerable effect on STH transmission and prevalence, and this potentially increased risk of STH recrudescence needs to be evaluated [59].

#### Metrics and cost-effectiveness thresholds

The wide range of effectiveness metrics used by the different studies hinders direct comparison of their results. This has been noted for other NTDs as well [50].

The ideal choice of metric for evaluating control strategies will often be the number of DALYs averted, as it allows the cost-effectiveness estimates to be directly compared to that of other healthcare interventions. This makes it possible to have standardised thresholds for policymakers, which class whether or not an intervention is cost-effective - which is rarely possible when reporting a disease specific cost per infection case averted. However, it is important to restate that, as discussed in the “The cost-effectiveness of control *versus* elimination” section, DALYs averted and incremental cost-effectiveness ratios may not reflect the true value of alternative interventions aimed at accelerating and sustaining elimination or disease eradication. In addition, DALYs are not without limitations, and their design contains inherent flaws that fail to acknowledge the implications of local context on disease burden [60], which is particularly important for NTDs which are most prevalent in poor populations. Furthermore, clinical LF has an impact on the quality of life for patients as well as their families, which is not fully captured by a DALY weight. It is also important to consider that due to a lack of data, features of the disease burden are ignored. For example, all of the current DALY estimates for LF assume it is not associated with any excess mortality (which could underestimate its burden). It is also worth noting that Ton et al. [61] found that accounting for the mental illness that can be experienced by LF patients and their caregivers significantly increased the DALY burden estimates related to LF. This has not currently been included in any the economic evaluations of LF control, which therefore underestimates its cost-effectiveness/cost-benefit. Non-filarial elephantiasis (podoconiosis) has also been found to be associated with depression [62].

There is debate and uncertainty surrounding the most appropriate cost per DALY averted thresholds for defining which interventions are classed as cost-effective [63, 64]. It should be noted that the thresholds established by the World Bank [18] are more conservative than the thresholds set by WHO-CHOICE [39] (a cost per DALY averted > 3 times the national gross domestic product (GDP) per capita = not cost-effective; between 1 and 3 times the national GDP per capita = “cost-effective”; and < 1 times the national GDP per capita = “very cost-effective”). However, these WHO thresholds are now widely considered to be too high [63–66] and are rarely used for NTD interventions. A recent analysis indicated that a cost per DALY averted threshold closer to ½ the national per capita GDP would be more appropriate for low-income countries [67]. Interestingly, a subsequent study used a threshold of US$ 200 per DALY averted to identify priority interventions for consideration in low-income countries [68].

#### Reporting standards for economic evaluations

Elements of the studies were not always clear, and at times important pieces of information were not reported. Moving forward it would be beneficial if studies were to adhere more to standardised guidelines (such as CHEERS [69]) regarding what should be reported within the manuscript.

### Evaluation of alternative interventions

Though we found five cost-effectiveness estimates relating to alternative strategies to the standard dual drug MDA strategy (Table 1), there are still notable research gaps in this area. In particular, the following are some key interventions that will require further economic evaluation in the future.

#### Anti-*Wolbachia* therapy and other novel drug treatments

A novel approach for treating LF involves using tetracycline antibiotics (such as doxycycline), to target the parasites *Wolbachia* endosymbionts which are essential for worm fertility and survival [70, 71]. A six-week course of doxycycline has been reported as a safe and well-tolerated treatment for LF, with significant activity against the adult worms [71]. Treatment also improves mild to moderate lymphoedema independent of ongoing infection [72]. An important benefit of this intervention is that it can also be used to treat onchocerciasis and is safe in loiasis co-endemic areas (as *L. loa* do not have any *Wolbachia*). One of the primary goals of the Anti-*Wolbachia* Consortium (A-WOL) is to identify drugs or regimens that reduce the period of treatment from weeks to days [71].

Other potential macrofilaricides should also be evaluated if they become available [73–79].

#### Triple drug administration

Triple drug administration with ivermectin, albendazole and DEC (IDA) has been shown to keep participants free of microfilariae for up to two years after treatment [80]. In contrast, within the same study over 90% of the control group (who received the standard dual drug therapy) tested positive for microfilaria after only one year [80]. This shows that IDA is a more effective treatment strategy and a potential method for accelerating transmission elimination (this is supported by mathematical modelling studies [81]). However, this strategy is not currently applicable to most of sub-Saharan Africa, as DEC is non-permissible for use in onchocerciasis endemic areas, and ivermectin is not recommended where intense loiasis transmission occurs [15]. Alternative approaches to manage these programmatic exceptions have been proposed [15, 44]. For example:

(i) A Test-to-Exclude from treatment strategy is currently being evaluated in loiasis-endemic areas [44]. However, were this strategy to be widely adopted, an increase in operational costs of the LF elimination strategy would be expected.
(ii) Pre-treatment with ivermectin in onchocerciasis endemic areas followed by the IDA regimen is also being considered (a “pretreat and treat” approach) [15]. Such an approach would have substantial benefits for LF elimination and, possibly, onchocerciasis elimination, but would likely also incur an increase in programmatic costs.

Although IDA has the potential to be a game changer for LF elimination, more research is required to determine if there is a safe and effective way to use it in co-endemic settings before it is approved for these areas [15]. In particular, the restrictions regarding the use of DEC in onchocerciasis-endemic areas would need to be addressed through robust and extensive studies showing that IDA can be used safely in these settings [15].

#### Vector control

The potential impact of vector control on LF transmission has been illustrated by several studies [82]. For example, a study in the Gambia, which found that even without MDA, LF transmission may have been interrupted through the extensive and long-term (decades) use of insecticide-treated nets for malaria control [83]. A malaria eradication campaign in the Solomon Islands was also found to result in the interruption of LF transmission in the absence of MDA [84]. In addition, Nsakashalo-Senkwe et al. [85] found a significant decline in LF transmission associated with the nationwide scale-up of insecticide-treated nets in Zambia. These studies highlight how the expansion of insecticide-treated nets for malaria control since 2000 [86], could have had a notable impact on LF transmission in some settings [87]. A more detailed review of the role of vector control in the GPELF is provided by Bockarie et al. [82].

Due to the long-life expectancy of the adult worms and the delay between infection and morbidity, the use of vector control as a standalone strategy would result in a lag before any significant effect on the prevalence of infection and morbidity is seen [88]. This finding is mainly because vector control programmes only reduce exposure to new infections and do not have a direct effect on the established infections within the host population. Although the established adult worms will die naturally within their hosts, this occurs slowly due to their long-life expectancy [88]. However, in combination with MDA, vector control could potentially be beneficial in accelerating progress to elimination, preventing transmission hotspots and reducing the risk of the re-establishment of the transmission cycle from imported cases [82, 87–89]. This indicates that in the context of economic evaluations, the true potential benefits of combining vector control with MDA are long-term - in contrast to additional short-term reductions in morbidity or infection. This means that economic evaluations of vector control would require a long-time horizon for the analysis and a model accounting for the possibility of elimination to capture its full long-term benefit.

It is noteworthy that the only study we identified evaluating the cost-effectiveness of integrating vector control with MDA (which found that it did not appear to be cost-effective in the investigated setting [37]) had only a five-year time horizon (Table 1). Due to this, the potential longer-term benefits of vector control were not necessarily fully captured.

In the context of further economic evaluations of vector control for LF, it is essential to note that its benefit will be highly dependent on the local species of vector. For example, bednets will not be effective in areas where the predominant vector species bites during the day. This highlights the importance of not overgeneralizing the results of studies and policy in this area. It is also important to consider issues relating to insecticide resistance and the additional benefits of vector control on other vector-borne diseases (such as dengue and malaria) [90]*.*

#### Diagnostics and surveillance strategies

As well as new interventions, we need to evaluate novel diagnostics and surveillance strategies. The importance of this research area is highlighted by a recent study which demonstrated resurgence of transmission six years after stopping MDA [91]. When considering new surveillance strategies, it is important to note the potential need to integrate surveillance for other NTDs (such as STH) [92, 93]. Only one of the studies [20] we identified explicitly considered the cost of post-MDA surveillance.

## Conclusions

LF occurs across a wide and diverse range of epidemiological settings, making it difficult to draw conclusions regarding the value of LF interventions as a whole from studies based in a single country or setting. Also, due to the different aims of the identified studies and the different approaches used, it can be difficult to directly compare the results of the different studies. However, overall this systematic review highlights that the WHO recommended strategies for LF elimination are consistently found to be cost-effective or cost-saving across a wide range of settings and assumptions. This finding has important implications for advocacy groups and potential funders. However, there are several important research gaps that need to be addressed as we move forward towards the 2020 milestones and beyond. These include the evaluation of alternative interventions (such as IDA, anti-*Wolbachia* therapy and vector control). Furthermore, elements of the studies were not always clear, and at times important pieces of methodological information were not reported. Moving forward it would be beneficial if studies adhered more to standardised guidelines for reporting cost-effectiveness analysis - allowing easier comparison of the different studies results.

## Additional file

Additional file 1: **Table S1.** Variation in the estimated wage/value of an agricultural worker. **Text.** Search terms for PubMed. **Table S2.** PRISMA checklist. (PDF 563 kb)

## Abbreviations

A-WOL: Anti-*Wolbachia* Consortium
DALYs: Disability-adjusted life years
DEC: Diethylcarbamazine
GDP: Gross domestic product
GPELF: Global Programme to Eliminate Lymphatic Filariasis
IDA: Triple drug administration with ivermectin, albendazole and DEC
LF: Lymphatic filariasis
MDA: Mass drug administration
NTD: Neglected tropical diseases
STAG: Strategic and Technical Advisory Group for NTDs
STH: Soil-transmitted helminths
WHO: World Health Organization
